# Genetics of helminth infections: Immune system response, insights into host-parasite interaction, and drug resistance

**DOI:** 10.5455/javar.2025.l879

**Published:** 2025-03-24

**Authors:** Mashael Abdullah Aldamigh

**Affiliations:** Department of Biology, College of Science, Majmaah University, Al-Majmaah, Saudi Arabia

**Keywords:** Helminthiases, genomics of helminthiases, host-parasite interaction, immune system response, drug resistance

## Abstract

Helminthiases, which are caused by parasitic helminths, have a big effect on global health, especially in places with few resources. They cause a lot of illness and put a lot of strain on society and the economy. Understanding the prevalence, transmission, and impact of helminthiases is crucial for effective control and prevention. Molecular population genetics has been pivotal in understanding helminth dynamics, including species identification, hybridization, and drug resistance. However, deeper insights require broader genetic datasets. Several genomes have been sequenced using genomic technologies, which has changed the way helminth researchers do their work and made it easier to compare genomes and find conserved genetic elements. Genetic factors of the host also affect susceptibility. Genome-wide association studies have found candidate genes that are connected to susceptibility or resistance. Helminth infections trigger Type 2 immune responses involving various immune cells, cytokines, and mediators. Recent discoveries show how non-immune cells like stromal, epithelial, and neural cells control these responses. Genetic differences between the host and the parasite affect how they interact. Helminths use immunomodulatory molecules to hide from immune surveillance. The concept of host disease tolerance, maintaining health despite infection, is gaining attention. The emergence of drug resistance poses a challenge, emphasizing the need to understand the genetic mechanisms underlying anthelmintic resistance. Genomic approaches offer promising avenues for interventions, including vaccine development and RNA interference. Challenges in helminth genetics research include genetic heterogeneity, limited sample sizes, and technical constraints. Using both functional genomics and multi-omics methods together can help us fully understand helminth genetics and plan effective treatments. Genomic studies have helped us learn more and find possible targets for interventions. To turn these findings into useful control measures, we need to do more research and work together.

## Introduction

As a whole, helminthiases, which are parasitic infections caused by nematodes, trematodes, and cestodes, are still a major health problem around the world. These infections affect millions of people worldwide, particularly in resource-limited settings, and have a profound impact on human health, leading to significant morbidity and socio-economic burden. Understanding the prevalence, transmission, and impact of helminthiases is crucial for effective control and prevention strategies. Helminthiases are widespread in various regions around the world, particularly in tropical and subtropical areas. Roundworms (*Ascaris *lumbricoides), whipworms (*Trichuris trichiura*), and hookworms (*Necator americanus *and *Ancylostoma duodenal*) are some of the soil-transmitted helminths (STHs) that more than 1.5 billion people around the world are affected by Montresor et al. [64]. Approximately 240 million people worldwide suffer from schistosomiasis, which is carried on by blood flukes (Schistosoma spp.). Sub-Saharan Africa, as well as several regions of South America, the Caribbean, and Asia, has the largest prevalence of the disease [14]. The transmission of helminthiases occurs through various routes, primarily associated with poor sanitation, inadequate hygiene practices, and environmental factors. Fecal-oral transmission is common for many helminth infections. Contaminated soil, water, or food serves as reservoirs for the infective stages of helminths, which are then ingested by susceptible individuals [1]. For example, STHs are commonly acquired by ingestion of eggs or larvae present in contaminated soil, whereas schistosomiasis is acquired through direct contact with freshwater bodies infested with cercariae released by snail intermediate hosts [1].

Understanding the genetic basis of helminthiases is crucial for unraveling the host-parasite interactions, transmission dynamics, drug resistance, and potential targets for intervention strategies. Molecular population genetics has been a highly productive area of research in understanding the population biology of helminth parasites over the past 40 years. Despite a slower adoption in the beginning compared to other fields, parasitologists have increasingly utilized molecular population genetic methods to explore the ecology, epidemiology/epizoology, and evolution of various parasitic helminths [2,61]. These methodologies have been particularly valuable in overcoming challenges associated with complex life cycles and the need for host dissection, enabling indirect insights into population biology [4–7].

By analyzing specific genetic regions or using a panel of genetic markers, such as mitochondria or ribosomal DNA regions, or codominant markers like microsatellites and allozymes, parasitologists have made significant strides in understanding helminth population dynamics. These molecular markers have been essential in uncovering hidden aspects of parasite studies, including cryptic species identification, hybridization, modes of reproduction, mating systems, effective population sizes (Ne), phylogeography, local transmission dynamics, and anthelmintic drug resistance [8,9]. Despite the progress made in parasite population genetics, the initial generation of studies required considerable time and financial investments, often limited to the use of a few targeted genetic loci.

While a handful of genetic markers can address many ecological questions, some areas of research still face limitations or difficulties. For example, drug resistance studies might have missed crucial loci due to a focus on specific candidate genes, historical demographic estimates may have been less precise [10,11], and conclusions about evolutionary history based on a single marker could be biased towards that locus rather than the entire species, and patterns of mito-nuclear discordance might not be sufficient to distinguish between incomplete lineage sorting and hybridization [12,13]. As a result, further advancements and the use of broader genetic datasets are needed to overcome these challenges and gain deeper insights into parasite populations.

### Helminth genomics

The advent of genomic technologies has revolutionized the study of helminths by providing insights into their genetic makeup and evolutionary relationships. Several helminth genomes have been sequenced, including those of human parasites such as *Ascaris lumbricoides*, *Schistosoma mansoni*, and *Taenia solium *[14–16]. These genomic resources have enabled comparative genomics and identified conserved genetic elements across different helminth species.

### Genetic determinants of helminth infection

Host genetic factors play a crucial role in determining susceptibility or resistance to helminth infections. Candidate genes and genetic variants linked to increased or decreased vulnerability to certain helminthiases have been found through genome-wide association studies (GWASs). For instance, a GWAS study on *Schistosoma mansoni* infection identified genetic variations in the IL-4 receptor gene associated with resistance to infection [18]. Similarly, variations in genes involved in innate immunity, such as TLRs and cytokines, have been linked to susceptibility to various helminth infections [19]. Inbred mice maintained in controlled, pathogen-free surroundings are typically used in studies examining the factors that influence host response during helminth infection; this method is widely used at research centers and academic institutions around the world.

Nevertheless, it is essential to consider that real-world helminth infections occur in diverse communities with varying lifestyles and significant genetic variations among individuals. Additionally, the intensity of helminth infection can differ significantly among people due to a combination of genetic and environmental factors [20]. Therefore, in this review, we investigate the influence of genetic and environmental diversities on the regulation of the immune response elicited by helminth infections, which results in the observed variations in responses among individuals. We also shed light on current research efforts and prospects for investigating the interplay between environment, genetics, and other factors that influence the variability observed in individuals during helminth infection.

### Helminth infection and the body’s immune response

Research on genetically altered mice with the C57BL/6 genetic background over the past few decades has considerably increased our understanding of Type 2 responses to helminth infections. The recruitment and accumulation of different innate immune cells, such as eosinophils, basophils, innate lymphoid cells, neutrophils, and alternatively activated macrophages, characterize these immune responses. Additionally, B cells, Th2, and T regulatory CD4 T cells from the adaptive immune system play crucial roles in these responses [21–26]. Type 2 and regulatory cytokines, as well as other mediators produced by these various cell types, play critical protective and regulatory roles during helminth-induced inflammation [27–29].

**Figure 1.**
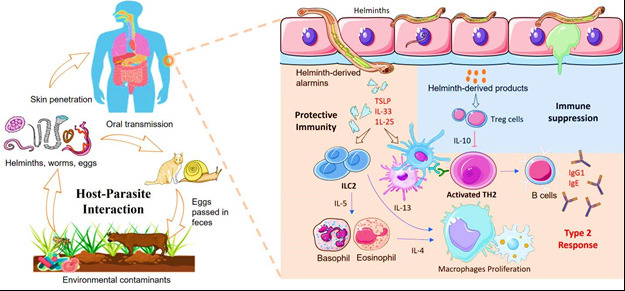
Graphical description of host-parasite interaction and body immune response.

Epithelial, neuronal, and stromal cells, which were previously neglected, play an important role in controlling type 2 immune responses during helminth infections, according to recent significant advances [17,30,31]. These non-immune cells can create bioactive mediators like cytokines and alarmins that interact with innate and adaptive immune cells to affect the overall helminth infection response (Fig. 1). Despite these developments, a thorough knowledge of the variations in these responses between individuals and the impact of host genetics and environmental interactions in free-living mammals is still lacking.

### Helminth infection and genetic variability

The ability to manipulate the genetics of mice has been instrumental in advancing our understanding of mammalian physiology. Due to its inbred origin, the C57BL/6 mouse strain has grown over time to become the preferred option for immunological research. This predilection has led to substantial advancements in our understanding of the basic processes that control the immune response during helminth infection. Researchers have been able to investigate several mediators and immune cell types involved in controlling the immune response to helminths by using genetically altered mouse models. With the addition of cell-specific knockouts, inducible fate mapping models, global knockouts, and transgenic mice, these models have advanced significantly. These methods have shown unique mechanisms controlling type 2 and immunoregulatory responses to helminths. Researchers use genetically identical mouse strains of similar age groups and, in some cases, the same sex to isolate and investigate cell types and immune mediators to assure consistency and reliability in these detailed experiments.

However, to examine the effect of genetic differences on helminth infection resistance, numerous studies have used inbred mouse strains [32]. For example, studies have shown that the BALB/c mouse strain is more vulnerable to the filarial worm Litomosoides sigmodontis than C57BL/6 or C57BL/10 mice [33–35]. The BALB/c strain, on the other hand, has demonstrated better resistance to other intestinal helminth parasites such as Trichuris muris [36–38] and Heligosomoides polygrus [39–41]. Several additional investigations have been conducted on various inbred strains with different helminth parasites. Although it is established that genetic diversity influences susceptibility and resistance to helminth infection in mouse models, it is still unknown exactly what mechanisms underlie these variations in the immune response.

Our knowledge of how genetic variation affects the primary and/or secondary sentinels of Type 2 inflammation [42–44], such as epithelial, stromal, and neuronal cells, is still limited. While some studies have investigated the involvement of various effector immune cells, cytokines, and immunoglobulins (Table 1), our understanding of how genetic variation affects genetic variation is still limited. These facets have not been fully investigated. For instance, in the field of epithelial cell biology, tuft cell responses in C57BL/6 and BALB/c mice have varied both under steady-state settings and in response to the protozoan parasite Trichomonas muris. However, after persistent infection with *H. polygyrus* at the height of parasite establishment, there were no significant differences in tuft cell response [45]. Variable inbred mouse strains may have variable tuft cell hyperplasia kinetics, with BALB/c animals possibly responding more robustly than C57BL/6 mice [46]. It is fundamental to use a variety of inbred mouse strains to study the part played by these sentinels in the disorder and outcome of helminth infection.

### Pathogenesis of helminth infection and genetic variability

When it comes to helminth infections, it is crucial to distinguish between the functions of genetic variation in resistance and pathogenesis. Although resistance and pathogenesis are related because individuals heavily infected often experience disease morbidity, the mechanisms that drive disease pathogenesis might not necessarily be the same as those responsible for parasite resistance. Our current knowledge of pathogenesis during helminth infections mainly comes from studies involving genetically modified mouse models. The pathways, cytokines, and other mediators that control the disease processes have been better understood because of these models. Additionally, by infecting inbred strains of mice and observing the differences in disease outcomes, we have gained further clues about the diversity of the immune response and the development of severe pathology.

The balance of cytokines present during an infection plays a crucial role in defining how pathogenesis develops and affects both tolerance and resistance to the infection. The release of alarmins such as IL-33, IL-25, and TSLP at epithelial barriers coincides with the detection of pathogen-associated molecular patterns by pattern recognition receptors shortly after infection [47,48]. This causes transcription factors to become active, such as *STAT*6 and *GATA*3, which then upregulate several genes, including receptors, cytokines, chemokines, and genes that control the generation of eicosanoids [49–51].

A greater number of effector cytokines are released because of the recruitment, accumulation, and differentiation of immune cells by cytokines, chemokines, and eicosanoids. By encouraging repair, differentiation, and the release of effector molecules from the epithelial barrier, these effector cytokines in turn aid in the removal of the worms [52–54]. Therefore, the initiation of anti-helminth immune responses as well as the pathophysiology of these responses are greatly influenced by cytokines, chemokines, eicosanoids, and other mediators.

### Host-parasite interactions

Helminths have evolved complex mechanisms to modulate host immune responses and establish chronic infections. Genetic variations in both host and parasite can influence the outcome of host-parasite interactions. For example, certain helminth species express immunomodulatory molecules that manipulate host immune responses, allowing them to evade immune surveillance and persist within the host [55]. Conversely, host genetic factors, such as variations in immune-related genes, can influence the intensity of the immune response and affect the susceptibility or resistance to helminth infections. Individuals living in areas with a high prevalence of helminth infections remain highly susceptible to re-infection despite repeated deworming treatment. Even though they are unable to resist different parasitic helminth species that have co-evolved with their hosts for thousands of years, it is astonishing that they rarely sustain major tissue damage. This suggests that mammals may have developed mechanisms to tolerate these infections. The detailed interaction has been illustrated in Figure 1.

Disease tolerance is a defense mechanism that promotes host health by limiting tissue damage caused by pathogens without directly targeting the pathogen burden. While disease tolerance has been well-documented in plant systems and is evolutionarily conserved, its exploration in mammals has only recently gained attention as a mode of host defense.

### Host disease tolerance: a defensive strategy

Girolamo Fracastoro proposed the germ theory in the 16th century, asserting that microorganisms caused communicable diseases. Louis Pasteur confirmed this theory three hundred years later, leading to significant progress in understanding immunity and the development of life-saving antibiotics. However, this theory initially overlooked the diverse roles of microbes within their hosts. Today, it is widely acknowledged that mammals have a symbiotic relationship with various bacterial, viral, and fungal species [56].

Despite the significant prevalence of helminths worldwide, epidemiological data from areas where they are common indicate that people and these parasites have developed a mutualistic relationship. To protect themselves against these parasites, humans have developed efficient defense systems, such as type 2 immunity, which has led to a low death rate. Except in extreme situations where symptoms may be caused by physical blockage rather than inflammation-induced tissue destruction, infections with some helminths, such as *A*.* lumbricoides* and *T*.* trichiura*, rarely result in clinical symptoms during the intestinal stage [1]. This indicates that humans and other animals have developed tolerance to these infections. Wild rats are also seen to have this resistance to helminth infections. 90% of wild mice were found to contain at least one roundworm, including *T*.* spiralis*, *H*.* polygyrus*, and *T*.* muris*, according to studies [38]. Repeated delivery of less than 40 *H. polygyrus* larvae to laboratory mice resulted in an asymptomatic chronic infection [3].

### Drug resistance

**Figure 2.**
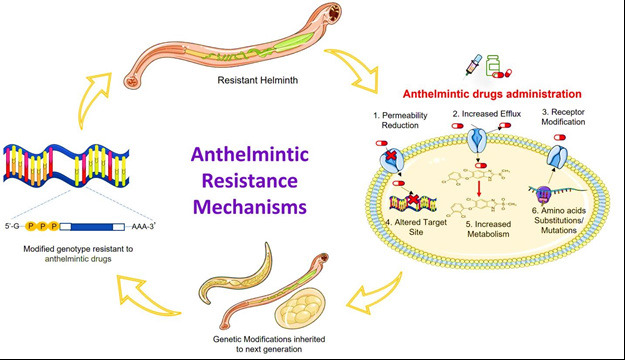
An overview of helminthic drug resistance.

The emergence of drug resistance poses a significant challenge in the treatment and control of helminthiases. Drug resistance in helminths develops and spreads mostly because of genetic causes. Studies have identified genetic mutations and mechanisms associated with resistance to commonly used anthelmintic drugs, such as benzimidazoles and ivermectin [57,58]. These genetic variations can confer reduced drug efficacy and hinder the success of control programs (Fig. 2).

### Genetic basis of drug resistance

Similar to various pathogens, parasitic helminths can develop resistance to drug treatments at a fast pace. To effectively combat this issue, it is crucial to comprehend the genetic mechanisms underlying anthelmintic drug resistance in parasitic nematodes. Understanding the spread of resistance is crucial in monitoring and devising strategies to enhance the effectiveness and sustainability of parasite control measures. Over 1 billion people, as well as numerous livestock and companion animals, require regular drug treatment to control infections caused by parasitic worms (helminths). However, the rapid and widespread evolution of resistance to anthelmintic drugs, especially in livestock-infective helminths, has become a significant issue. In various locations, specific drug classes have lost their effectiveness, and certain farms are now harboring parasites resistant to all major drug classes [59].

In Europe, helminths in livestock lead to annual production losses of €686 million, out of which €38 million is attributed to anthelmintic resistance [60]. While research has predominantly focused on livestock parasites [62], drug resistance has also become a major concern in the treatment of the dog heartworm Dirofilaria immitis and the dog hookworm Ancylostoma caninum (a clade III species related to human filarial nematodes) in the United States [63,64]. The same drugs used to control related helminths that infect humans are targeted by widespread preventive chemotherapy programs worldwide. Although the emergence of anthelmintic resistance is less established in helminths infecting humans, it is likely to have significant socioeconomic and welfare impacts on infected individuals, potentially hindering the progress made in eliminating helminths as a public health concern in the upcoming decade. The widespread emergence of anthelmintic resistance shares similarities with the global crisis of antimicrobial resistance [65].

### Genomic approaches for helminth control

Genomic studies offer promising avenues for developing new interventions and control strategies against helminthiases. Comparative genomics can help identify essential genes and metabolic pathways specific to helminths, which can serve as potential drug targets. Additionally, omics approaches, including transcriptomics and proteomics, provide insights into the molecular interactions between helminths and their hosts, aiding in the identification of key molecules for vaccine development or immunotherapies [66].

By leveraging the power of genomics, researchers can gain valuable insights into the biology of these parasitic worms and develop innovative strategies for their control and management. Here we would like to enlist some important areas that can be used as resourceful reservoirs for control of helminth via genomic studies.

#### Genome sequencing

The complete sequencing of helminth genomes provides a wealth of information about their genetic makeup, evolution, and potential vulnerabilities. Comparative genomics is a powerful tool that allows researchers to compare the genomes of different organisms. This can be used to identify genes that are unique to a particular parasite and which are therefore essential for its survival. These genes could be potential drug targets, as targeting them could effectively kill the parasite.

#### Transcriptomics

Transcriptomic studies involve analyzing the complete set of RNA transcripts produced by the parasite at a particular stage of its life cycle or under specific conditions. This approach helps researchers understand which genes are active at different stages of development or during drug exposure, aiding in the identification of drug-resistant strains.

#### Proteomics

Proteomics involves the study of the entire set of proteins expressed by the helminth. By analyzing the proteome, researchers can gain insights into the proteins that are crucial for the parasite’s survival and identify potential targets for therapeutic intervention.

#### Metagenomics

Metagenomics is the study of the genetic material of entire communities of microorganisms, extracted directly from environmental samples. This can be used to assess the diversity of helminth populations in each environment and to understand the potential for these parasites to be transmitted to humans.

#### Pharmacogenomics

Pharmacogenomic studies focus on understanding the genetic basis of drug response in helminths. By identifying genetic markers associated with drug resistance, researchers can develop better strategies for administering anthelmintic drugs.

#### RNA interference (RNAi)

RNAi is a promising approach to knock down specific gene expression in helminths. By introducing small interfering RNAs (siRNAs) that target essential parasite genes, researchers can inhibit their growth and development.

#### Vaccine development

Genomic information can aid in the identification of potential vaccine candidates by highlighting antigens that are specific to the parasite and elicit a protective immune response. By combining these genomic approaches with other disciplines such as bioinformatics, systems biology, and functional genomics, researchers can gain a comprehensive understanding of helminth biology and develop more targeted and effective strategies for helminth control and treatment.

### Future perspective

Despite significant advancements, there are several challenges and gaps in understanding the genetic basis of helminthiases. Genetic heterogeneity, limited sample sizes, and technical constraints pose challenges for genetic studies in helminths. Further research is needed to elucidate the genetic basis of host susceptibility, parasite virulence, and the mechanisms underlying drug resistance. Integration of multi-omics approaches, functional genomics, and systems biology can provide a more comprehensive understanding of helminth genetics and inform targeted interventions.

## Conclusion

The genetic basis of helminthiases plays a vital role in disease susceptibility, host-parasite interactions, drug resistance, and control strategies. Genomic studies have expanded our knowledge of helminth genetics and provided avenues for identifying potential targets for intervention. Continued research and collaboration are essential to unravel the complex genetic mechanisms underlying helminthiases and translate these findings into effective control measures.

## List of abbreviations

DNA, Deoxyribonucleic acid; GWAS, A genome-wide association study; ILC2, innate lymphoid cells; PRRs, pattern recognition receptors; RNA, Ribonucleic acid; RNAi, RNA interference; STHs, the soil-transmitted helminths; STAT6, Signal transducer and activator of transcription 6; siRNAs, small interfering RNAs; WHO, World Health Organization.
